# P-1129. Exploring Cost Savings through Reduced Sedation for Magnetic Resonance Imaging in Pediatric Osteomyelitis Diagnosis

**DOI:** 10.1093/ofid/ofae631.1316

**Published:** 2025-01-29

**Authors:** Lauren Sumner, Rachel Downey, Sarmistha Bhaduri Hauger, Lynn Thoreson, John Williams, Brian Kaufman

**Affiliations:** Dell Children's Medical Center, Austin, TX; Dell Children's Medical Center of Central Texas, Austin, Texas; Dell Children's Medical Center; Dell Medical School at the University of Texas at Austin, Austin, Texas; University of Texas at Austin Dell Medical School, Austin, Texas; Dell Children's Medical Center, Austin, TX; Dell Medical School at the University of Texas at Austin, Austin, Texas

## Abstract

**Background:**

Magnetic resonance imaging (MRI) is essential for the diagnostic workup of pediatric osteoarticular infection. In the pediatric population, sedation is often required to mitigate motion artifact and improve imaging quality, which contributes to cost, resource utilization, and risk to the patient. In 2021, our hospital implemented an evidence-based update to the pediatric osteomyelitis guideline adjusting MRI imaging sequences, which reduced sedation age requirements from 8 years to 3 years of age. This change was initiated in September 2021. This study evaluates economic implications of sedation reduction strategies in pediatric osteomyelitis imaging.

**Figure 1.**
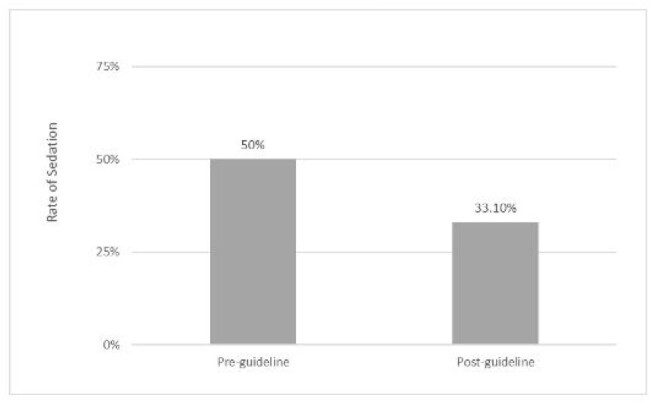
Pre vs Post Guideline Sedation Rates.

**Methods:**

This is a retrospective chart review study of pediatric patients evaluated for bone and joint infection by MRI at a free standing 299 bed hospital in Central Texas. Cases were pulled by MRI indication according to the hospital’s osteomyelitis guideline inclusion criteria between January 1, 2020 and December 31, 2023. Previously healthy children age 6 months to 18 years with physical exam suggestive of acute hematogenous osteomyelitis or septic joint and less than 14 days of symptoms were included. Cases were compared by year pre-guideline (2020-2021) and post-guideline (2022-2023) and reviewed for age, sex, need for sedation, and diagnosis after MRI. Cost savings estimates were based on an average per case cost of sedation. The cost of sedation was determined by hospital charges.

**Figure 2.**
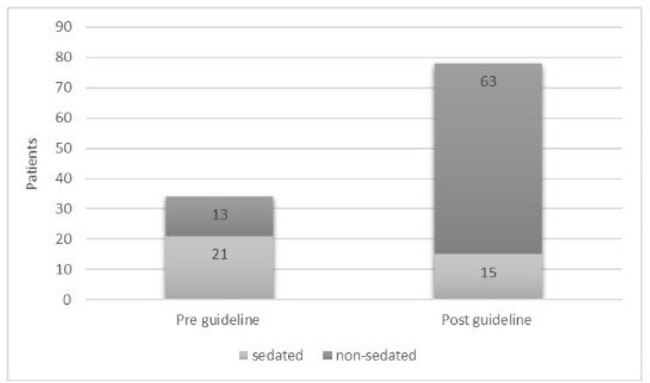
Pre & Post Guideline Sedated vs Non-Sedated Patients.

**Results:**

279 patients were included, (98 pre guideline change; 181 post guideline change). 61.3% male, 38.7% female, median age 6, IQR 6.5. A statistically significant reduction in sedation usage was observed after guideline implementation (50%-33%, p=0.0087) (fig 1). Among patients 3-8 years of age, 21 of 34 patients (62%) pre-guideline and 15 of 78 patients (19%) post- guideline received sedation (p< 0.001) (fig 2). An average estimated cost savings of $270,215 per year was found. There was no increase in repeat MRI rates seen with the implementation of the age requirement change.

**Conclusion:**

Lowering the required age for sedation allows for individualized assessment of sedation needs in the 3-8 age group. This leads to improved patient care through decreased patient risk, reduction in overall rate of sedation and results in cost savings.

**Disclosures:**

**Brian Kaufman, MD**, Johnson & Johnson: Advisor/Consultant

